# The utility of universal urinary drug screening in chronic pain management

**DOI:** 10.1080/24740527.2018.1425980

**Published:** 2018-02-08

**Authors:** Luke K. Wiseman, Mary E. Lynch

**Affiliations:** a Faculty of Medicine, Dalhousie University, Halifax, Nova Scotia, Canada; b Department of Anesthesia, Pain Management & Perioperative Medicine, Dalhousie University and Nova Scotia Health Authority, Halifax, Nova Scotia, Canada; c Department of Pharmacology, Dalhousie University and Nova Scotia Health Authority, Halifax, Nova Scotia, Canada; d Department of Psychiatry, Dalhousie University and Nova Scotia Health Authority, Halifax, Nova Scotia, Canada

**Keywords:** chronic pain, urinary drug screening, toxicology

## Abstract

**Background:**

A recent systematic review found few studies that assessed the value of urinary drug screening (UDS) in the management of chronic pain. The Pain Management Unit in Halifax, Nova Scotia, has recently implemented tandem mass spectrometry (TMS) UDS for all new patients.

**Aims:**

To study the prevalence of unexpected TMS UDS results at a hospital-based chronic pain center, to assess which drugs are most likely to contribute to an unexpected result and to assess the clinical utilization of unexpected results by pain physicians.

**Methods:**

From June 2014 to June 2016, a total of 664 patients with chronic non-cancer pain (CNCP) were seen for initial consult. Charts were reviewed and used to create a database containing sex, age, UDS result, physician, and medication/illicit drug history. For all unexpected UDS results, an interview was conducted with the treating physician to determine its clinical implications.

**Results:**

For the general pain specialists, the overall percentage of patients with an unexpected UDS result was 16.67%. Excluding codeine, at most 4.47% of patients tested unexpectedly positive for a strong opioid. Although eight out of nine physicians found UDS helpful in general, only 29.58% of unexpected results were helpful in the management of their patients and directly influenced their care.

**Conclusions:**

The prevalence of an unexpected UDS result in patients with CNCP is significant. Most physicians agree that UDS is helpful but in only a limited number of cases did the unexpected result provide helpful information that significantly influenced patient care.

## RÉSUMÉ

**Contexte**: Une revue systématique récente a démontré que peu d’études évaluaient la valeur du dépistage urinaire de drogue dans la prise en charge de la douleur chronique. L’Unité de prise en charge de la douleur d’Halifax, en Nouvelle-Écosse, a récement instauré le dépistage urinaire de drogue par spectrométrie de masse en tandem pour tous ses nouveaux patients.

**But**: Éudier la prévalence de résultats inattendus lors du dépistage urinaire de drogue par spectométrie de masse en tandem dans un centre de la douleur chronique en milieu hospitalier, afin d’évaluer quelle drogues sont les plus susceptibles de donner lieu à un résultat inattendu et à la fois évaluer l’utilisation clinique des résultats inattendus par les médecins spécialisés dans le traitement de la douleur.

**Méthodes**: De juin 2014 à juin 2016, 664 patients souffrant de douleur chronique non cancéreuse ont été vus pour une consultation initiale. Leurs dossiers ont été étudiés et utilisés pour créer une base de données contenant leur sexe, leur âge, le résultat obtenu lors du dépistage urinaire de drogue, leur médecin traitant et leurs antécédents en matière de médication ou de drogues illicites. Pour tous les résultats de dépistage urinaire de drogue inattendus, une entrevue a eu lieu avec le médecin traitant afin d’en déterminer les implications cliniques.

**Résultats**: Pour les spécialistes de la douleur générale, le pourcentage global de patients ayant obtenu un résultat inattendu lors du dépistage urinaire de drogue était de 16,67 %. À l’exclusion de la codéine, tout au plus 4,47% des patients ont obtenu un résultat positif inattendu pour un opioïde puissant. Bien que huit médecins sur neuf aient trouvé le dépistage urinaire de drogue utile en général, seulement 29,58% des résultats inattendus ont été utiles dans la prise en charge de leurs patients et ont influencé directements les soins prodigués.

**Conclusions**: La prévalence d’un résultat inattendu lors du dépistage urinaire de drogue chez les patients souffrant de douleur chronique non cancéreuse est élevée. La plupart des médecins sont d’accord pour dire que le dépistage urinaire de drogue est utile, mais que le résultat inattendu a fourni de l’information utile ayant influencé les soins aux patients de manière significative seulement dans un nombre limité de cas.

## Introduction

Opioids are a critical tool in the management of acute and cancer related pain, as well as a subpopulation of appropriately selected patients with chronic non-cancer pain (CNCP). The use of opioids in CNCP has become a controversial topic due to the potential for opioid related harms. The risk of side effects, drug diversion, and abuse of opioids has been well documented.^1–4^ Rates of addiction during chronic opioid therapy vary in the literature, with a Cochrane database review reporting a rate of 0.27%^5^ and a recent systematic review reporting a rate between 8% to 12%.^6^ There is currently a great deal of pressure on physicians prescribing opioids due to recent guidelines and regulatory changes. Media and others have fueled a harsh opioid prescribing climate by citing opioid deaths without clarifying the role of illicit fentanyl from China.^7^ The challenge that faces the prescriber is to provide appropriate treatment to the individuals who need it while minimizing risk. This can be addressed by using appropriate screening, well-supervised medication trials, prescription monitoring programs, and possibly prescribing abuse-deterrent formulations.^8^

Urinary drug screening (UDS) is one method to verify medication history and can be used as an adherence and risk assessment tool. It can reinforce healthy behavioral change, increase patient and physician communication, and ensure safe medication use by detecting behaviors suggestive of nonmedical use.^9–11^ The Canadian National Opioid guidelines recognize that baseline UDS may be useful, but there are few studies that look at UDS for risk mitigation.^12^ A recent systematic review investigated the efficacy of UDS and found few studies that assessed the value of UDS in pain management.^13^

As with any test, clinical judgment is required when interpreting the result. The clinician must understand the test ordered, the patient’s pretest probability, the purpose of obtaining the test, and the false positive and negative likelihoods. Applying these principles when ordering a test and having an accurate testing method will reduce mislabeling a patient based on a false result.

A UDS result can either be expected or unexpected. A UDS result can be unexpectedly positive if the patient does not disclose his or her full medication and illicit drug history. A UDS result can be unexpectedly negative due to an incomplete medication history, nonadherence, or drug diversion. It is important to have accurate testing to allow confidence in a UDS result as part of the patient care plan.

Three main types of UDS tests can be implemented in clinical practice. Point-of-care testing can be achieved by enzyme immunoassay but is limited with regards to the number of drugs that can be tested and is less sensitive and specific than laboratory testing. Laboratory immunoassay provides improved accuracy but often misses semisynthetic and synthetic opioids. Tandem mass spectrometry (TMS) allows for accurate testing of semisynthetic and synthetic opioids and lower screening cutoff values.^14^ The high accuracy of TMS UDS testing reduces the chances of false positives and negatives, which can improve the clinician’s confidence with the test result.^15^ Caution is required when interpreting TMS UDS results because metabolites can be detected. Therefore, it is important to have a good understanding of opioid and benzodiazepine metabolites. For instance, knowing that codeine can be metabolized to morphine, hydrocodone, and hydromorphone is critical when analyzing TMS UDS results. Although TMS has less cross-reactivity than immunoassay, they both cannot differentiate between l and d isomers.^16^ This may lead to false positive amphetamine results when the patient is taking medication such as selegiline, levorphanol, or dextromethorphan.^17^

Within the current literature, the rates of unexpected UDS results vary and not every study looked at how these results influence patient care. The number of patients with CNCP who had an unexpected UDS result varied from 8.8% to 41.3%, with results primarily consisting of unexpected marijuana and cocaine use.^18–30^ This large variability may be attributed to the type of UDS test used and how the physicians selected their patients for UDS. One study showed that UDS may reduce illicit drug use. After 14 visits using UDS there was a decrease in illicit drug use from 23% to 9%. However, it was unclear whether this was due to UDS or patients not continuing to see their physician after the UDS result.^31^

Since 2012, the Pain Management Unit (PMU) in Halifax, Nova Scotia, has been conducting UDS on all new patients referred for consultation. Most referrals to the clinic are from family physicians but some are from specialists or other pain physicians within the clinic. The pain physician completes a new assessment on the patient and, as per the PMU protocol, a UDS test is completed. The PMU is in a tertiary care hospital that focuses on chronic pain management through a multidisciplinary team. UDS is done universally on all new patients to destigmatize UDS. The medications that are screened for include cannabinoids, cocaine, benzodiazepines, opioids, and other psychoactive substances that could be abused.

A previous study done at the PMU looked at UDS using laboratory immunoassay testing “Emit II plus” on all new patients from February 2012 to July 2013.^18^ The study found that 5.49% of patients had at least one unexpected UDS result based on their self-medication report. A physician survey within this study found that immunoassay testing was effective in patient management in 50% of unexpected test results. One of the main concerns of UDS by the physicians was the inadequacy of immunoassay for opioid detection. This may lead to false negatives that could underrepresent the true discordance between UDS results and patient reporting.

Since April 2014, the PMU has adopted the more accurate TMS UDS. The present study examines the prevalence of unexpected TMS UDS results, the drugs most likely to contribute to an unexpected result, and how these results are being utilized by pain physicians.

## Methods

A retrospective chart review was completed on all new patients referred for consultation at the PMU in Halifax, Nova Scotia, from June 2014 to June 2016. This study was approved by the Nova Scotia Health Authority Research Ethics Board on April 1, 2016.

A total of 684 patients were assessed at the PMU from June 2014 to June 2016. At the PMU, there are nine general pain specialists and one pain/addictions specialist. The UDS results were separated based on the type of specialist. The charts of all new patients with CNCP were reviewed and used to create a database containing patient sex, age, physician, UDS toxicology result, and medication/illicit drug history.

The principal investigator reviewed the available information in the patient’s paper chart and electronic record in order to document current medications and history of illicit drug use. Information reviewed included patient self-reported medication history, nursing clinical notes, physician clinical and dictation notes, notes written on the UDS result, physician referral notes, prescription medication history faxed by pharmacy, and electronic Nova Scotia Prescription Drug Monitoring Program records. The clinical information available varied from chart to chart, but there was always sufficient information to determine the patient’s current medication and illicit drug use history.

A UDS result was classified as unexpected if it did not match the patient’s up-to-date illicit drug history, medication history, or known metabolites. The UDS result was classified by the pain physician or principal investigator with review by the treating physician. The clinical judgment of the treating physician would ultimately determine whether the result was expected or unexpected.

The frequency of patients with CNCP not providing an accurate prescription drug and illicit drug use report was determined by comparing the number of unexpected results to the total number of UDS tests completed. The rate for general pain specialists and the pain/addictions specialist could be determined by looking at the treating physician’s interpretation on the UDS result. Drug classes and individual opioids tested for on TMS UDS were also analyzed. Drug metabolites were taken into consideration when determining whether the result was unexpected and when determining how many drugs were present on the UDS result. A literature search was conducted and laboratory medicine was consulted to determine opioid and benzodiazepine metabolites that could be detected by UDS.^32^ Laboratory medicine was also consulted on the price of TMS UDS vs. immunoassay UDS.

In cases where there was an unexpected result, further information was collected from the treating physician with a five-question quantitative (yes/no) and qualitative questionnaire (Appendix). Informed consent was obtained from each physician. The quantitative data were presented as a descriptive percentage. The qualitative data were analyzed using a straightforward general inductive approach.^33^ The questionnaire transcripts were studied and coded repeatedly and emerging categories were developed. The categories clarified how unexpected results were or were not helpful and how these results were utilized in the management of patients at the PMU. Similarities and differences across helpful and nonhelpful unexpected UDS results were explored. Barriers to UDS at the PMU were also identified.

## Results

Six hundred eighty-four new patients were examined at the PMU from June 1, 2014, to June 30, 2016. Of the 684 patients, 20 had cancer pain and were excluded from the study. Of the 664 CNCP patients, 438 had UDS toxicology results. Of the available UDS results, 24 were from the pain/addictions specialist and 414 were from general pain specialists. Although efforts are made to complete UDS on all new patients, this is a relatively new routine and in practice not all patients are screened. A comparison of demographics between the group with available toxicology and the group without is presented in Table 1.

**Table 1.** Demographics of patients with CNCP for group with toxicology available vs. group without (*n* = 664).

	Toxicology available (*n* = 438)	Toxicology not available (*n* = 226)
Mean age (years)	50.19	56.19
% 80 + (*n* = 37)	2.05	12.39
% 60–79 (*n* = 165)	23.29	27.88
% 40–59 (*n* = 328)	52.05	44.25
% 20–39 (*n* = 118)	19.63	14.16
% 0–19 (*n* = 16)	2.97	1.33
% Male	42.92	36.73
% Female	57.08	63.27

CNCP = Chronic non-cancer pain.

UDS results were positive for at least one drug in 60.96% of cases (Table 2). The most commonly detected drugs were opioids, followed by cannabinoids and benzodiazepines. Few patients tested positive for cocaine, amphetamines, and barbiturates, and no patient had a positive phencyclidine result (Table 3).

**Table 2.** Number of drugs testing positive on UDS.^a^

Number of drugs on UDS		% Toxicology available (*n* = 438)
0	171	39.04
1	148	33.79
2	73	16.67
3	31	7.08
4	12	2.74
5 or more	3	0.68
2 or more opioids	45	
2 or more BDZ	13	

^a^Drug metabolites were not counted if the parent drug was present.

UDS = urinary drug screening; BDZ = benzodiazepines.

**Table 3.** Positive UDS results in respective drug classes.

Drug	Positive UDS result	% of toxicology available with positive UDS result (*n* = 438)
Amphetamine	4	0.91
Benzodiazepine	90	20.55
Barbiturate	1	0.23
Cannabinoid	106	24.20
Cocaine	6	1.37
Opioid	186	42.47
Phencyclidine	0	0

UDS = urinary drug screening.

In the group with toxicology available for general pain specialists, the highest rates of unexpected UDS results were seen with opioids, benzodiazepines, and cannabinoids (Table 4). The percentage of UDS results for general pain specialists’ patients with at least one unexpected result (positive and negative) was 16.67% (69/414). The rate for testing unexpectedly positive for a drug was 14.25% (59/414).

**Table 4.** Unexpected UDS results in respective drug classes for general pain specialists.^a^

Drug	Unexpected positive on UDS	Unexpected negative on UDS	Total number of unexpected UDS results	% of unexpected UDS results (*n* = 69) classified as unexpected for drug	% of UDS results (*n* = 414) classified as unexpected for drug
Amphetamine	0	1	1	1.45	0.24
Barbiturate	1	1	2	2.90	0.48
Benzodiazepine	12	5	16	23.19	3.86
Cannabinoid	14	1	15	21.74	3.62
Cocaine	5	0	5	7.25	1.21
Opioid	27	15	41	59.42	9.90
Phencyclidine	0	0	0	0	0
Total	59	23	69		16.67

^a^In the opioid drug class, there was one UDS result that had an opioid test unexpectedly positive and a different opioid test unexpectedly negative. Though this result had both an unexpected positive and negative, it still only counts as one patient having an unexpected UDS result. Therefore, the sum of unexpected positives and negatives in the opioid drug class does not equal the total number of unexpected opioid UDS results. This was also the case for the benzodiazepine drug class. There were 13 unexpected UDS results with two drugs that were unexpected. Therefore, the total number of unexpected UDS results does not equal the total number of unexpectedly positive and negative drugs on UDS.

UDS = urinary drug screening.

A total of 54 opioids/metabolites were unexpected for general pain specialists, with codeine, morphine, hydromorphone, and oxycodone contributing the most (Table 5). Unlike Tables 2, 3, and 4, metabolites are included in the counts of Table 5 because there was no way to determine which opioid the patient was consuming. For instance, a patient testing unexpectedly positive for codeine and morphine may be taking just codeine or both codeine and morphine. In this case, the patient would be considered to have two unexpectedly positive opioid/metabolite counts. These results show the percentage of the unexpected opioid UDS results in Table 4 that are contributed by a weak opioid (i.e., codeine) or a strong opioid.

**Table 5.** Unexpected UDS results for individual opioid/metabolite(s) for general pain specialists.

Opioid/metabolite	Unexpected positive on UDS	Unexpected negative on UDS	Unexpected positive or negative on UDS	% of unexpected opioid and metabolite counts on UDS (*n* = 54) contributed by opioid/metabolite
Codeine	17	4	21	38.89
Hydrocodone	1	0	1	1.85
Hydromorphone	5	5	10	18.52
Methadone	2	0	2	3.70
Morphine	10	3	13	24.07
Oxycodone	2	5	7	12.96
Total	37	17	54	100

UDS = urinary drug screening.

In the group with toxicology available for the pain/addictions specialist, unexpected opioids and benzodiazepines were detected at a rate of 33.33% (8/24) and 29.17% (7/24), respectively.

The rate for testing unexpectedly positive for opioids and benzodiazepines was 20.83% (5/24) and 29.17% (7/24), respectively. The percentage of pain/addiction specialist’s patients with at least one unexpected result was 50% (12/24). Ten opioids/metabolites were unexpected, with codeine accounting for 40% of unexpected opioids and morphine, oxycodone, and hydromorphone accounting for 30%, 20%, and 10%, respectively.

Most unexpected UDS results consisted of only one unexpected drug, but 22.22% (18/81) had multiple unexpected drugs. For example, a patient testing unexpectedly positive for cocaine and an opioid would be classified as two unexpected drugs. Metabolites were taken into consideration when classifying the number of unexpected drugs found on a UDS result. For example, if a UDS result was unexpectedly positive for codeine and morphine, then it would be considered as only one unexpected opioid result because morphine is a metabolite of codeine. However, if the result was unexpectedly positive for oxycodone and hydromorphone, then it would be considered as two unexpected opioids because they are not metabolites of one another; 6.17% (5/81) of unexpected UDS results had two unexpected opioids.

The price for universal UDS at the PMU decreased from $93.60 to $71.11 per sample by switching from laboratory immunoassay to TMS. Therefore, because 438 UDS tests were completed over the study period, a cost savings of $9,850.62 was achieved when considering cost of tests only. Laboratory medicine purchased two TMS generators using capital money at an estimated cost of $421,172 during the time TMS UDS was implemented in Halifax. The cost for setup and capital equipment of TMS vs. laboratory has an important role when considering the overall cost of TMS. The equipment for TMS was purchased using capital money, so it did not have a direct effect on the cost for individual tests.

### Questionnaire results

During the study, there were ten physicians at the PMU. One of the general pain specialists was unable to conduct the interview, so nine of the ten physicians were interviewed regarding 71 of the 81 unexpected results. In addition, 88.89% (8/9) of the physicians found that UDS was helpful in the care of their patients in general. Physicians answered yes to “Did you find that UDS information has been helpful in the care of this patient?” 64.79% (46/71) of the time.

#### Question: If you did find UDS helpful, will you please describe why and how it may have influenced the care of your patient?

The 46 unexpected UDS results that were helpful in the care of the patient were further classified based on how the result was said to have influenced patient care. Six categories emerged. These were changes in medication prescribing, patient education, referral/follow-up with another health care provider, follow-up UDS test, unaltered clinical decision making, or patient not seen after initial UDS.

Of all the helpful UDS results, 17.39% (8/46) led to changes in prescribing of medication. This ranged from tapering medications, limiting narcotics and controlled drugs substances, and changing pain medications to nonopioid options. For instance, one physician said, “I avoided increasing his dose of opioids and stuck to using neuropathic pain medications.”

Furthermore, 10.87% (5/46) of helpful UDS results led to an education session with the patient about the result. This included checking the accuracy of the patient’s self-reported medication history, informing patients of proper medication use, and educating patients of adverse effects of the medication(s). One physician stated, “Simply informing patient of findings is helpful even if they deny. Advised patient of aberrant behaviors and adverse effects of taking prescribed medication that was not prescribed to them.”

Furthermore, 10.87% (5/46) of helpful UDS results led to a follow-up with another health care provider. This included collaborative prescribing practices with another specialist, referral to an addictions specialist, and follow-up discussion with family physician. One physician wrote, “I spoke to his psychiatrist about some of these issues and we decided to taper some of his medications as a result.”

In addition, 6.52% (3/46) of helpful UDS results led to a follow-up UDS test. The follow-up UDS test was done to inform proper medication management. One physician recalled, “In this particular patient I did not change my treatment plan. I just did further samples and if they had another positive we would change dispense to weekly.”

The physician’s clinical decision making was not altered in 39.13% (18/46) of helpful UDS results. These were prescribers that answered “no” to “In what way did the helpful UDS result influence the care of your patient?” In these cases, the UDS information that the physician gained was helpful but did not result in a significant change in patient care. The UDS result allowed the physician to get a complete medication history. One physician stated, “Patient apparently not taking as much Tylenol # 3 as he says, which is likely a good thing for this patient. History is not reliable due to cognitive dysfunction. No effect on management of patient.”

Furthermore, 17.39% (8/46) of patients with helpful UDS results did not return to the PMU after the initial consult. Therefore, the pain physician could not discuss the result with the patient. One physician said, “Patient was not seen since UDS result, so effect on patient care not evident.”

Many physicians also agreed that expected UDS results can be helpful in the management of patients with CNCP. They found that using this information in conjunction with history was a valuable tool in an addiction risk assessment.

#### Question: If you did not find UDS helpful, will you describe why?

The 25 unexpected UDS results that were not helpful in the care of the individual patient were further classified based on why they were not helpful. Six categories emerged. These were unexpected cannabinoid detection, missed UDS result, nonadherence, nonprescription treatment plan, unaltered clinical decision making, or patient not seen after initial UDS.

First, 28% (7/25) of nonhelpful UDS results were unhelpful because of unexpected cannabinoid detection. Physicians indicated that they were not overly concerned with the presence of cannabinoids in their patients. One physician described, “I don’t pay a lot of attention to cannabis use because it is so widespread in our patients.” The UDS results show that 24.20% of patients with CNCP used cannabis at the PMU.

In addition, 12% (3/25) of nonhelpful UDS results were unhelpful because the result was missed. The missed UDS result was from either not recognizing that the UDS result was unexpected or not seeing the UDS result in the chart. One physician stated, “I did not see the unexpected result. Therefore, results were not acted on.”

Furthermore, 16% (4/25) of nonhelpful UDS results were unhelpful because nonadherence was suspected by the physician. These were cases where it was clear in the chart that the patient was taking regular dosing of the medication but screened negative. Reasons for nonadherence indicated by the physicians included financial constraints, no regular family doctor for refill prescriptions, or patient forgetfulness. One physician described, “The patient has no family doctor and opioid prescription is haphazard. Therefore, does not change my level of concern.”

Moreover, 24% (6/25) of nonhelpful UDS results were unhelpful because the physician was undertaking a nonprescription treatment plan to manage the patient’s pain. Many patients at the clinic underwent nonpharmacological therapies, including attendance at the pain self-management group, referrals to physiotherapy, or various types of nerve blocks to manage their pain. Physicians indicated that they were not planning on prescribing opioids or benzodiazepines to these patients, so it would not alter their management. One result was an unexpected negative amphetamine, which the physician did not prescribe in his practice. Another physician recalled, “I only assessed this patient for a nerve block long-term. Referred by another pain doctor.”

In addition, 12% (3/25) of nonhelpful UDS results were unhelpful because the physician’s clinical decision making was not altered by the result. The strategy that the physician had in place was not going to be influenced based on the UDS result. One patient had CNCP but also had cancer that was unrelated to the pain. Another physician said, “Did not influence clinical decision making. Patient suspected to be taking Tylenol # 1.”

Finally, 8% (2/25) of nonhelpful UDS results were unhelpful because the patient was not seen after the initial UDS.

#### Barriers identified

Barriers identified were time constraints and nonoptimal workflow. One physician stated that UDS adds 10 minutes to each patient assessment and minimally influences patient care. Workflow was brought up by multiple physicians. One physician indicated that having the UDS test one to two weeks before consultation would be better so that the results could be used during the initial assessment. This was evident throughout the questionnaires, because ten out of the 71 patients with an unexpected UDS result were not seen again in the clinic after the initial assessment. In additon, physicians indicated that having the UDS result with the patient’s paper chart would be beneficial when assessing whether the result was expected or not. This was evident throughout the questionnaires, because three out of the 71 unexpected results were missed.

## Discussion

For the general pain specialists, the overall percentage of patients with an unexpected UDS result (positive and negative) was 16.67%. This rate is similar to the rates of unexpected UDS results found in previous studies, which is between 8.8% and 41.3%.^18–30^

A similar study, conducted in a hospital-based pain management program, examined unexpected UDS using gas chromatography/mass spectrometry.^20^ Results showed that 20% of patients had an illicit substance in their urine, most commonly cannabis and cocaine. They also found that 10.2% of UDS showed the absence of a prescribed opioid. However, this study looked specifically at patients who were prescribed opioids and did not conduct universal UDS on all new patients. In the present study, many patients at the PMU were not using opioids. Furthermore, 57.53% of UDS results did not detect an opioid and 39.04% of patients had a negative toxicology report.

The overall percentage of patients with an unexpected UDS result was 50% for the pain/addictions specialist. For both specialists, opioids and benzodiazepines accounted for most of the unexpected UDS results. These are important drugs to consider when prescribing pain medications due to increased risk of sedation. In addition, there is evidence that benzodiazepines increase opioid toxicity and risk of overdose. One study found that most opioid overdoses involve multiple drugs in addition to opioids, with benzodiazepines and alcohol being the most common.^34^

For all UDS results completed by general pain specialists, 9.90% of the results were unexpected for an opioid (positive and negative) and 6.52% (27/414) were unexpectedly positive for an opioid. When calculating which individual opioid and metabolite contributed to the 9.90%, codeine contributed the most at 38.89% (21/54), followed by morphine at 24.07% (13/54). However, when interpreting these results, it is important to consider the metabolite(s). For example, morphine is a metabolite of codeine, so it was not always possible to know whether the patient was taking only codeine or codeine and morphine. However, the patient would be taking codeine in either situation because it is not a metabolite of any other opioid. Therefore, 38.89% of all unexpected opioids related to codeine may be an underestimate. Because at least 38.89% of the total 9.90% was due to a weak opioid (i.e., codeine), at most 6.05% of all UDS results for general pain specialists were unexpected (positive and negative) for a strong opioid. Similarly, in the general pain specialists group, because at least 31.48% (17/54) of the unexpected positive opioid results (6.52%) were due to codeine, at most 4.47% of all UDS results were unexpectedly positive for a strong opioid.

Currently in Nova Scotia, acetaminophen with codeine preparations do not require a prescription if the preparation contains no more than 8 mg or its equivalent of codeine per solid dosage unit or more than 20 mg or its equivalent of codeine phosphate per 30 ml in a liquid preparation.^35^ The readily available access to codeine may have contributed to it being the most common unexpected drug on UDS. Patients may feel that it is not important to disclose nonprescription medications to their health care provider. One study showed that physicians ask about over-the-counter drug use in 37% of patient encounters and only 58% of patients told their physicians about their over-the-counter use.^36^

The most common way that an unexpected UDS result influenced care was through changes in medication prescribing followed by patient education, follow-up with another health care provider, and follow-up UDS test. There is evidence that some of these actions can reduce substance abuse. One study found that a combination of UDS, treatment agreements, pill counts, and education reduced substance abuse by 50%.^37,38^

Although this study shows that most unexpected UDS results do not directly influence patient care, it did not evaluate expected results. Expected results can provide meaningful information to a physician. For example, an expected UDS result that is negative for illicit substances can increase the prescriber’s confidence when prescribing an opioid. This may have contributed to the eight out of nine physicians finding universal UDS helpful in their practice.

When unexpected UDS results were reported to be not helpful, it was mostly due to cannabinoid detection, followed by nonprescription treatment plan, suspected nonadherence, missed UDS result, unaltered clinical decision making, and patient not seen after initial consult. This highlights that sometimes even unexpected UDS results do not provide information that is helpful in patient management; however, these results could have been helpful to another physician. Immunoassay point-of-care testing is one method that allows immediate UDS results, which would have been beneficial in ten out of 71 patients with unexpected UDS results because they were not seen after initial consult. However, this method may result in increased mislabeling of patients due to the inaccuracy of immunoassay testing and may also miss unexpected results, especially those for semisynthetic opioids.

Although the unexpected presence of cannabinoids was the most common reason an unexpected UDS result was not helpful, it is important for physicians to be aware of its use for a number of reasons. This includes the potential for additive sedation with other medications, adverse effects, and to assure that the patient is not using cannabis to escape or “chemically cope” with stress.^39,40^ The rate of cannabinoid use in patients with chronic pain is quite high, approaching one quarter of patients in this study, and because most are using for pain control, the concern regarding cannabinoid detection is lower.

Two major barriers to UDS were identified at the PMU. These were time constraints and nonoptimal workflow. Having patient records readily available when assessing the UDS result can be important when determining whether the result is expected or unexpected. This was evident because three out of the 71 unexpected results were missed. Performing UDS before the initial consult and having the results at the initial visit may be beneficial. This would have been helpful in 14.08% (10/71) of patients with unexpected results because they were not seen after the initial visit.

One of the major limitations of this study was selection bias. Although universal UDS is the standard of care at the PMU, only 65.96% (438/664) of all new patients had UDS completed. The discrepancy was small, but males were more likely to have UDS completed than females. In addition, UDS was less likely to be completed in older patients, starting at the age of 60. Other patient factors such as socioeconomic status and diagnosis that were not collected could have contributed to selection bias. This bias could have overestimated the rate of unexpected results. This was a retrospective study, so no conclusions could be made on how universal TMS UDS changes practice. In addition, during the physician questionnaires, the management of an unexpected result was sometimes difficult to determine and often involved the physician looking retrospectively in the patient chart. Interview questionnaires were not conducted on one of the ten pain physicians at the PMU who had relocated his practice. Therefore, all opinions on unexpected UDS results during the time of the study were not captured. However, a 90% response rate captured major themes regarding UDS helpfulness, utilization, and barriers.

The results of the present study provide useful information regarding this academic tertiary care clinic but may not be generalizable to all pain clinics. The PMU in Halifax receives patients via referral and is a hospital-based pain clinic using TMS UDS. Many clinics across Canada may not have adopted TMS UDS in their practice, some clinics may not receive patients through referral or be hospital based, and the patient population in Halifax may be different from that of other pain clinics elsewhere. In addition, the latter may have important implications when considering positive morphine results because heroin can be metabolized into morphine.^32^ Heroin use is historically low in Nova Scotia and no patients reported heroin use in this study; however, this will vary depending on location of the pain clinic.^41^

This study only looked at one screening tool for risk mitigation. Although physicians in the study performed a detailed history to screen for risk of abuse, no validated screening questionnaire tool was used. It would be beneficial for future research to include validated screening tools for substance use disorders. It would also be valuable to have fentanyl added to the list of drugs tested on TMS UDS because illicit fentanyl is an increasing cause of opioid deaths in the United States and Canada.

This study is the first to have a defined rate of unexpected UDS results using TMS on new patients presenting to a hospital-based pain clinic. It is also the first study to examine how these results are being utilized by pain physicians. The rate of unexpected UDS results is common in patients with CNCP. Unexpected UDS results are utilized by pain physicians to influence patient care in many ways, but most of these results are either unhelpful or do not alter clinical decision making.

## Conclusion

For the general pain specialist group, one in six patients had an unexpected UDS result. Of the 414 UDS tests completed on general pain specialists’ patients, at most 6.05% were unexpected for a strong opioid when including both positive and negative unexpected results and 4.47% were unexpectedly positive for a strong opioid. Furthermore, 1.21% of all UDS results were unexpected for the presence of cocaine. Benzodiazepines, codeine, cannabinoids, barbiturates, and amphetamines made up the remainder of unexpected results. Most physicians agreed that UDS is helpful, but in only a limited number of cases did the unexpected result provide helpful information that significantly influenced patient care. When UDS impacted patient care, it provided information to improve collaborative practices and patient–physician communication. It was also used as a method to guide further testing and prescribing.

## Disclosure of Interest

Luke Wiseman has no conflicts of interest to declare. Mary Lynch has no conflicts of interest to declare.

## Supplementary Material

1425980--Supplemental_Material.docx

## Appendix

**Physician questionnaire following patient’s unexpected urinary drug screening (UDS)**

Patient name: ______________________________

Attending clinician: ______________________________

1. Do you find that UDS information is helpful in the care of your patients in general?
  a. Yes
  b. No
2. Did you find that UDS information has been helpful in the care of this patient?
  a. Yes
  b. No
3. If you did find UDS helpful, will you please describe:
  a. Why?
  b. In what way it may have influenced the care of your patient?
4. If you did not find UDS helpful, will you please describe why?
5. Please include any other comments you may have on the topic of UDS, such as your thoughts about UDS in the management of chronic pain.
