# Supplementary material for: The utility of universal urinary drug screening in chronic pain management
Source: Can J Pain. 2018 Feb 8;2(1):37–47. doi: 10.1080/24740527.2018.1425980 (PMC8730562; doi:10.1080/24740527.2018.1425980)
Supplement: 1425980--Supplemental_Material.docx [file UCJP_A_1425980_SM6162.docx]

**Supplementary Tables:**

Table 1: Drug/metabolite screening cut-off concentrations using laboratory immunoassay at the PMU.

| **Drug/metabolite** | **Screening cut-off concentration (ng/ml)** |
| --- | --- |
| Amphetamine and Methamphetamine | 1000 |
| Barbiturate | 200 |
| Benzodiazepine | 200 |
| Cannabinoid | 50 |
| Cocaine metabolite | 300 |
| Codeine and Morphine | 2000 |
| Phencyclidine | 25 |

PMU = Pain Management Unit.

Table 2: Drug/metabolite screening cut-off concentrations using TMS at the PMU.

| **Substance class** | **Substance** | **Screening cutoff**  **(ng/mL)** |
| --- | --- | --- |
| Amphetamines | Amphetamine | 250 |
|  | Methamphetamine | 250 |
|  | MDMA | 250 |
|  | MDA | 250 |
| Benzodiazipines | Diazepam | 100 |
|  | Nordiazepam | 100 |
|  | Oxazepam | 100 |
|  | Temazepam | 100 |
|  | Triazolam | 100 |
|  | Alpha-hydroxy-triazolam | 100 |
|  | Alprazolam | 100 |
|  | Alpha-hydroxy-alprazolam | 100 |
|  | Clonazepam | 100 |
|  | 7-Amino-clonazepam | 100 |
|  | Lorazepam | 100 |
| Cannabinoid Metabolite | THC carboxylic acid | 50 |
| Cocaine Metabolite | Benzoylecgonine | 100 |
| Methadone | Methadone | 100 |
|  | EDDP | 100 |
| Opioids | Codeine | 300 |
|  | Morphine | 300 |
|  | Oxycodone | 100 |
|  | Hydromorphone | 100 |
|  | Hydrocodone | 100 |
| Phencylidine | Phencyclidine | 25 |

TMS = Tandem Mass Spectrometry; PMU = Pain Management Unit; MDMA = Methylenedioxymethylamphetamine; MDA = Methylenedioxyamphetamine; THC = Tetrahydrocannabinol; EDDP = 2-ethylidene-1,5-dimethyl-3,3- diphenylpyrrolidine

(Methadone metabolite).
